# Comparative analysis of avian poxvirus genomes, including a novel poxvirus from lesser flamingos (*Phoenicopterus minor*), highlights the lack of conservation of the central region

**DOI:** 10.1186/s12864-017-4315-0

**Published:** 2017-12-06

**Authors:** Olivia Carulei, Nicola Douglass, Anna-Lise Williamson

**Affiliations:** 1 Division of Medical Virology, Department of Pathology, Faculty of Health Sciences, University of Cape Town, Cape Town, South Africa; 2 Institute of Infectious Disease and Molecular Medicine, University of Cape Town, Cape Town, South Africa; 3 National Health Laboratory Service, Cape Town, South Africa

**Keywords:** Poxvirus, Avipoxvirus, Flamingopox, Genome sequence

## Abstract

**Background:**

Avian poxviruses are important pathogens of both wild and domestic birds. To date, seven isolates from subclades A and B and one from proposed subclade E, have had their genomes completely sequenced. The genomes of these isolates have been shown to exhibit typical poxvirus genome characteristics with conserved central regions and more variable terminal regions. Infection with avian poxviruses (APVs) has been reported in three species of captive flamingo, as well as a free-living, lesser flamingo at Kamfers dam, near Kimberley, South Africa. This study was undertaken to further characterise this virus which may have long term effects on this important and vulnerable, breeding population.

**Results:**

Gene content and synteny as well as percentage identities between conserved orthologues was compared between Flamingopox virus (FGPV) and the other sequenced APV genomes. Dotplot comparisons revealed major differences in central regions that have been thought to be conserved. Further analysis revealed five regions of difference, of differing lengths, spread across the central, conserved regions of the various genomes. Although individual gene identities at the nucleotide level did not vary greatly, gene content and synteny between isolates/species at these identified regions were more divergent than expected.

**Conclusion:**

Basic comparative genomics revealed the expected similarities in genome architecture but an in depth, comparative, analysis showed all avian poxvirus genomes to differ from other poxvirus genomes in fundamental and unexpected ways. The reasons for these large genomic rearrangements in regions of the genome that were thought to be relatively conserved are yet to be elucidated. Sequencing and analysis of further avian poxvirus genomes will help characterise this complex genus of poxviruses.

**Electronic supplementary material:**

The online version of this article (10.1186/s12864-017-4315-0) contains supplementary material, which is available to authorized users.

## Background

Avian poxviruses are important pathogens of both wild and domestic birds. In domestic poultry, avian poxvirus infection can cause significant economic losses due to transient decrease in egg production, impaired fertility, reduced growth in young birds and increased mortality [1]. In wild bird populations, including endangered and endemic species, poxvirus infection may lead to secondary bacterial or fungal infections, decrease ability to care for young, and affect vision and/or the ability to feed making them prone to predation [2–4].

As of 2007, poxvirus infections had been reported in 278 species of wild and domestic birds from 70 families and 20 orders [5, 6]. Since then, the total number of avian species reported to be infected with a poxvirus has risen to at least 329 across 76 families and it is likely that many more species are susceptible (Unpublished data).

Genetic, phylogenetic and genomic analyses are increasingly being used to classify and characterise members of this large genus of viruses. To date, isolates from a chicken (*Gallus gallus*) (FWPV and Fp9) [7, 8], canary (*Serinus canaria*) (CNPV) [9], African penguin (*Spheniscus demersus*) (PEPV) [10], feral pigeon (*Columba livia*) (FeP2) [10], turkey (*Melleagris gallopavo*) (TKPV) [11] and two Pacific shearwaters (*Ardenna carneipes*) (SWPV-1); (*Ardenna pacificus*) (SWPV-2) [12], have had their genomes fully sequenced. Because of the relative lack of complete genome sequences of avian poxviruses, construction of phylogenies has to date relied on single gene analyses, with the P4b gene (fwpv167; cnpv240; vacv A3L) [13], one of the 49 genes conserved in all poxviruses, being the most commonly used [14–20]. These analyses have shown that the *Avipoxvirus* genus is divided into 3 clades, A (Fowlpox like viruses), B (Canarypox like viruses) and C (Parrotpox (PRPV) like viruses) as well as two proposed clades, D (Quailpox virus (QPV)) and E (Turkeypox virus(TKPV)). Clades A and B are further divided into several subclades which differ slightly in their composition depending on the genetic locus used for analysis and as such, are still being resolved.

Gene content and synteny have also been used to help elucidate evolutionary relationships between poxviruses. Alignment and comparison of the central genomic regions of viruses from eight *Chordopoxvirinae* (ChPV) genera (using the Vaccinia Virus (VACV) genome as the reference) showed that Molluscum contagiosum virus and FWPV were most divergent, encoding 40 and 33 unique genes in their central genomic regions respectively. In contrast, myxoma virus (MYXV), Yaba-like disease virus (YLDV), lumpy skin disease virus (LSDV) and swinepox virus (SWPV) contained three or less unique genes in this region. In terms of gene order, FWPV also showed major differences with large blocks of genes being translocated and/or inverted compared to VACV and the other ChPVs [21]. These findings are in accordance with what has been shown through phylogenetic analysis.

Poxvirus infections have been documented in four species of flamingo to date. The first documented case occurred in Chilean flamingos (*Phoenicopterus chilensis*) that were housed at a zoo in Hino City, Tokyo, Japan [22]. Two separate cases of infections in American flamingos (*Phoenicopterus ruber*) have been reported with the first occurring in a bird housed at the National Zoological Park in Washington DC [23]. In this case, a 4.5 kb *Hin*dIII fragment ranging from the equivalent of fwpv193–203, was reported to show 99.7% nucleotide identity to an isolate from an Andean condor (*Vultur gryphus*) which groups in clade B phylogenetically. A second case was reported in a young American flamingo housed at the Lisbon zoo. Phylogenetic analysis based on P4b and the CNPV 186–187 fragment showed this isolate to group in clade B2 with the highest identity to isolates from various species of bustard [24]. Another case occurred at a zoo in Japan but the infection was noted in two, young Greater flamingos (*Phoenicopterus roseus*). Based on analysis of the P4b gene this isolate was shown to group with two isolates from pigeons (PPV-B7 and CVL950), also in clade B2. All of the above cases occurred in captive flamingos [25].

Documented cases of poxvirus infections in birds in South Africa date back to the early 1960’s when infections were noted in Cape turtle doves (*Streptopelia capicola*) and a Cape thrush (*Turdus olivaceus*) [26]. Infections were later noted in ostriches (*Struthio camelus australis*) [27], an African penguin (penguinpox virus (PEPV) - *Spheniscus demersus*) [10, 17, 20, 28], a feral pigeon (pigeonpox virus (FeP2) - *Columba livia*) [10, 20], and a speckled (rock) pigeon (*Columba guinea*) [29].

The flamingopox virus (FGPVKD09) isolate further characterised in this study, was obtained during a poxvirus outbreak in 2008, that occurred in a permanent, breeding population of lesser flamingos (*Phoenicopterus minor*), living at Kamfers Dam, a perennial wetland near Kimberley, South Africa. All lesions seen in this population were of the cutaneous form (present on the legs and faces) and regressed over time showing little effect to their overall health [30]. Approximately 30% of the juvenile population was estimated to have developed lesions over the observation period from January to June 2008. The sample analysed was taken from a lesion near the tibiotarsal joint of a juvenile flamingo which was euthanised and examined further. Phylogenetic analysis based on the alignment of partial P4b nucleotide sequences showed that this isolate grouped in subclade A3 with 99–100% nt identity to the other A3 isolates from various species [18, 20, 30]. Due to this similarity, it was suggested that the flamingos were infected with a virus that was already in circulation in wild birds. This was the first reported case of poxvirus infection in free-living flamingos as well as the first reported case of a flamingo infected by a clade A virus.

Flamingos are gregarious in nature and therefore vulnerable to infectious disease. Due to the declining population and human induced threats to the already small number of breeding sites, the lesser flamingo is listed as near-threatened in both South Africa and internationally. This study was undertaken to further characterise this virus which may have long term effects on this important, breeding population.

## Methods

### Virus isolation

The Flamingopox virus (FGPV) sample was collected on post mortem from flamingo at Kamfers Dam by Dr. David Zimmermann and Dr. Mark Anderson as part of an investigation into the cause of the dermal lesions. The sample was donated by Dr. Emily Lane of National Zoological Gardens as scab tissue and stored at −20 °C until further processing. A portion of the scab was diced with a scalpel and added to a Dounce homogeniser in 1 ml of McIlvains buffer (pH 7.4) containing penicillin (500 U/ml), streptomycin (100 μg/ml) and fungin (1 μg/ml). The homogenate was centrifuged at 800 rpm for 5 mins and the supernatant used for inoculation onto the chorioallantoic membranes (CAMs) of embryonated hens’ eggs as described previously [31].

### DNA sequencing

DNA was extracted as described previously [10], and sent to the Central Analytical Facility (CAF) at the University of Stellenbosch, in Stellenbosch, South Africa for full genome sequencing. DNA was sheared ultrasonically using the Covaris S2 sample preparation system (Covaris Inc., USA). One 316 chip was used followed by use of one half of a 318 chip on the Ion Torrent Personal Genome Machine (PGM) (Life Technologies), according to the manufacturer’s instructions.

Basic quality control was performed by Anelda van der Walt (CAF, University of Stellenbosch, Stellenbosch, South Africa) using Torrent Suite software (version 3.2.1). Reads were trimmed of adaptor sequences and further trimmed if average base quality values (Q value) were <25 with window size = 11. Reads were discarded if read length was <50 nt and filtered to remove polyclonal reads. All reads that passed the above quality control filters were mapped to the chicken genome (*Gallus gallus* WASHUC2) using Newbler 2.6 and BLASTed to the chicken genome using CLC Genomics Workbench 4.7.1 (Qiagen) to filter out reads of host origin. Unmapped reads were used as input data for de novo assembly using the CLC Genomics Workbench 4.7.1.

### DNA analysis

CLC Genomics Workbench 4.7.1 was used for all analysis unless otherwise stated. Open reading frames (ORFs) longer than 90 nt with a methionine start codon (ATG) were identified. These ORFs were annotated as potential genes and numbered from left to right if alignment to the NCBI nr database using BLASTn and/or BLASTp and/or BLASTx gave BLAST expect values of E ≤ 1e-5. ORFs were annotated as described by Hendrickson et al., [32]. ORFs were annotated as intact (I) if the 5′ end is intact and the ORF is ≥80% the length of the closest homologue. If the 5′ end is intact but the ORF is <80% the length of the closest homologue, it was annotated as truncated (T). If the 5′ end is not intact the ORF was annotated as a fragment (F). If the ORF is ≥20% the length of the closest homologue it was annotated as extended (E) if the 5′ and 3′ ends were intact or as extended at the 5′ or 3′ end. Expression studies and functional analysis would be needed to determine whether fragmented and truncated ORFs are expressed and/or functional. The left most nucleotide was nominated as base 1, as the ITRs and terminal hairpin loops were not resolved. The FGPV sequence can be accessed from Genbank with accession number MF678796.

Dotplots were created using Gepard software, with word length = 10 [33]. Pairwise and multiple sequence alignments were created using MAFFT version 7, with default settings. Genome sequences of each of the fully sequenced avian poxvirus genomes (FWPV - AF198100; Fp9 - AJ581527; CNPV - AY318871; FeP2 - KJ801920; PEPV - KJ859677; TKPV - KP728110; SWPV1 - KX857216; SWPV2 - KX857215) were analysed to determine the number of conserved ORFs and copy numbers of gene family proteins. VACV (strain Copenhagen) - M35027 was also used in dotplot analysis.

The PEPV and FeP2 gene annotations in Genbank have been changed relative to the annotations found in the journal article describing the sequences [10]. When referring to PEPV and FeP2 ORFs in this study, the original annotations from the publication are used which are referred to in Genbank as “old locus tag”.

## Results

Ion torrent sequencing on the 316 chip resulted in 3,745,381 reads with a mean read length of 180 bp. After performing quality control (QC) and filtering reads of chicken origin, 1,309,385 (35%) poxvirus specific reads remained. On the 318 chip, 2,148,517 reads were generated with a mean read length of 251 bp. Only 570,143 (27%) remained after QC and filtering of reads of host origin. Read assembly in CLC Genomics workbench resulted in one contiguous sequence of 293,130 bp with an average of 1090× coverage. Basic genome statistics are shown in Table 1 below. The A + T content across the whole sequenced region was found to be 70.5% and to contain 285 potential ORFs encoding proteins ranging from 37 to 1984 amino acids in length, representing a coding density of 91% (Table 1). Relative to their closest orthologues, 259 ORFs have been annotated as intact, 21 as truncated and/or fragmented, and 4 as extended. One ORF, fgpv256 showed no similarity to any ORFs in Genbank but was identified to have a P-type ATPase motif and was therefore annotated as a hypothetical ORF. All FGPV ORFs have been listed and compared to their closest orthologues in an additional table (Additional file 1).

**Table 1:** Genome statistics of FGPV compared with each of the fully sequenced avian poxvirus genomes

Statistic	FWPV	FP9	FGPV	PEPV	FeP2	TKPV	SWPV1	SWPV2	CNPV
Length (kbp)	289	266	293	307	282	189	327	351	360
A + T (%)	69.1	69.2	70.5	70.5	70.5	70.2	72.4	69.8	69.6
# of ORFs	260	244	285	285	271	171	310	312	328

**Fig. 1:**
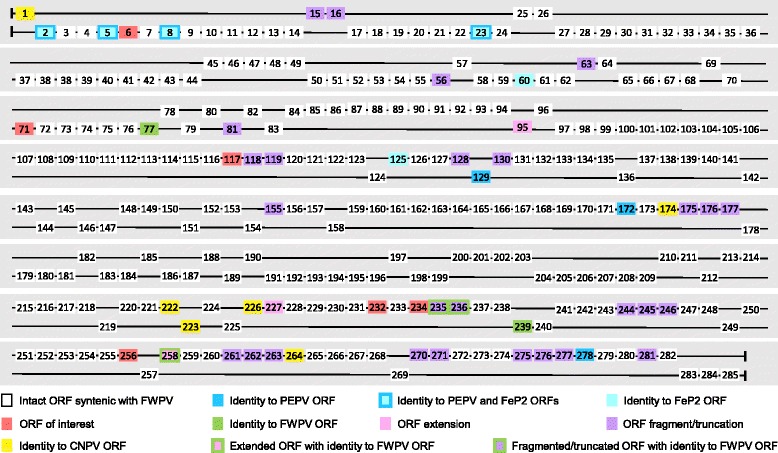
ORF schematic of the FGPV genome. The genome is depicted as double stranded, with ORFs shown as coloured blocks (not to scale), numbered from left to right. ORFs transcribed from left to right are depicted above and those transcribed from right to left depicted below

Comparison of the FGPV genome to other avian poxvirus, clade A genomes shows the central region to be relatively highly conserved in gene content and synteny while the terminal regions are more variable. Several ORFs in the terminal regions of the FGPV genome show greater nucleotide identity to CNPV ORFs than FWPV or other clade A ORFs and many are also truncated or fragmented compared to their closest orthologues (Fig. 1). Of the 25 FGPV ORFs noted to be truncated, fragmented or extended, eight (33%) encode hypothetical proteins, and 12 (46%) encode proteins belonging to gene families (ankyrin repeat, CC-chemokine and V-type Ig domain). ORFs of interest have been highlighted (Fig. 1, red blocks) that have either been identified in poxviruses for the first time (fgpv 232 and 256), appear to be novel members of gene families (fgpv117) or were previously identified and noted in other avian poxvirus genomes (fgpv006, fgpv071, fgpv234) (Table 2).

**Table 2:** Table of ORFs of interest identified in the FGPV genome as well as their location in eight other avian poxvirus genomes if present

ORF	FWPV	FGPV	PEPV	FeP2	TKPV	SWPV1	SWPV2	CNPV	Notes
IL-10	–	006	014	014	–	–	014	018	Found in similar genomic locations
UBQ	Frag.	071	074	Frag.	–	086	091	096	Found in the same genomic location
ANK	–	117	–	–	–	–	–	–	Ankyrin repeat family with limited (30%) identity to avipoxvirus ANKs
C-type lectin	–	232	–	–	–	–	–	–	C-type lectin / Brevican core protein/ NK receptor like
C7L	–	234	231	223	159	–	–	–	Host range gene thought to have been restricted to orthopoxviruses until 2014 [10]
P-type ATPase	–	256	–	–	–	–	–	–	No significant identity to known ORFs in Genbank

### Brevican core protein

FGPV encodes an orthologue fragment (fgpv232) of a C-type lectin with similarity to brevican core proteins encoded by eukaryotes. Amino acid identity is relatively low at 30–40%. In humans this protein is involved in nervous system development. To our knowledge, C-type lectins of this variety have not been identified in any viral species to date.

### Ankyrin repeat family

The FGPV genome was noted to have significantly more ankyrin repeat family proteins than what has been reported in other clade A avian poxviruses but if truncated and fragmented ORFs are assumed to be non-functional and therefore excluded, the number of ankyrin repeat family proteins decreases to levels previously noted in other, clade A, avian poxvirus genomes (FWPV and PEPV). The large number of disrupted ankyrin repeat proteins found in the FGPV genome could be due to gradual loss of these ORFs that were once the result of genomic accordion gene expansions. In the left hand, terminal region are four ORFs containing ankyrin repeats (fgpv002, fgpv005, fgpv008 and fgpv0023) that are only found in the genomes of South African isolates and five of the six FGPV ORFs that are homologues of ORFs only found in CNPV, are found in the terminal regions of the FGPV genome. FGPV also contains an ORF encoding an ankyrin repeat protein (fgpv275) with similarity to a serine/threonine protein phosphatase from various species including trichomonas vaginalis, and various insects and birds but not to any poxvirus ankyrin repeat proteins in Genbank. It is possible that this ORF was horizontally transferred from a host at some point in the evolutionary history of this virus.

### IL-10

FGPV encodes an IL-10 like protein (fgpv006) with identity to homologues found in the CNPV, PEPV and Fep2 genomes. This ORF is found in the same location as the copies found in PEPV and Fep2, between ORF equivalents pepv13-pepv15 and fep14-fep16. This region is highly conserved between the three African isolates.

### Ubiquitin

FGPV encodes an ubiquitin homologue (fgpv071) at the same genomic location as the CNPV and PEPV homologues, that shows 100% amino acid identity to homologues found in eukaryotes.

### C7L

Like PEPV, FeP2 and TKPV, FGPV contains an orthologue of orthopoxvirus C7L (fgpv234) which is found in an equivalent genome position between orthologues of fwpv216 and fwpv217. These orthologues are highly conserved with 96–98% aa identity. Although there is no ORF present, the equivalent region in the FWPV genome shows 67–68% nt identity to the above ORFs suggesting that FWPV may have once contained a C7L orthologue.

**Fig. 2:**
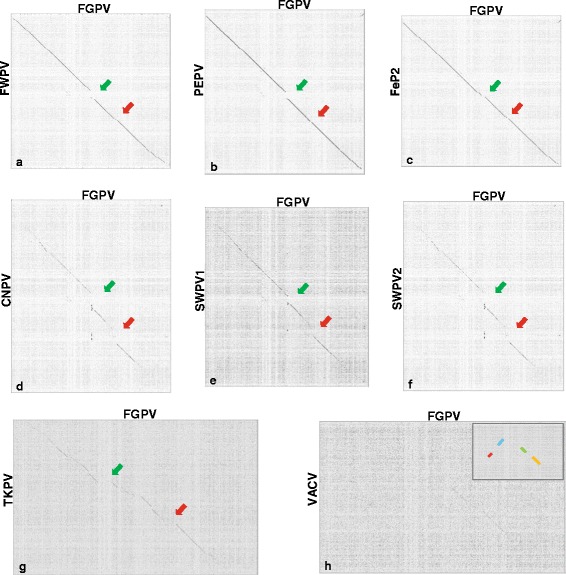
Dotplots of the FGPV genome (x axis) vs other sequenced avian poxvirus genomes (y axis). **a**) FGPV vs FWPV **b**) FGPV vs PEPV **c)** FGPV vs FeP2 **d**) FGPV vs CNPV **e**) FGPV vs SWPV1 **f**) FGPV vs SWPV2 **g**) FGPV vs TKPV **h**) FGPV vs VACV (Copenhagen) H inset) Conserved areas of the 2H) dotplot highlighted in colours corresponding to Fig. 5. Green arrows indicate the first region of difference (fwpv114–126) and red arrows indicate the second region of difference (fwpv146–165). Plots are not to scale. Window size = 10

**Fig. 3:**
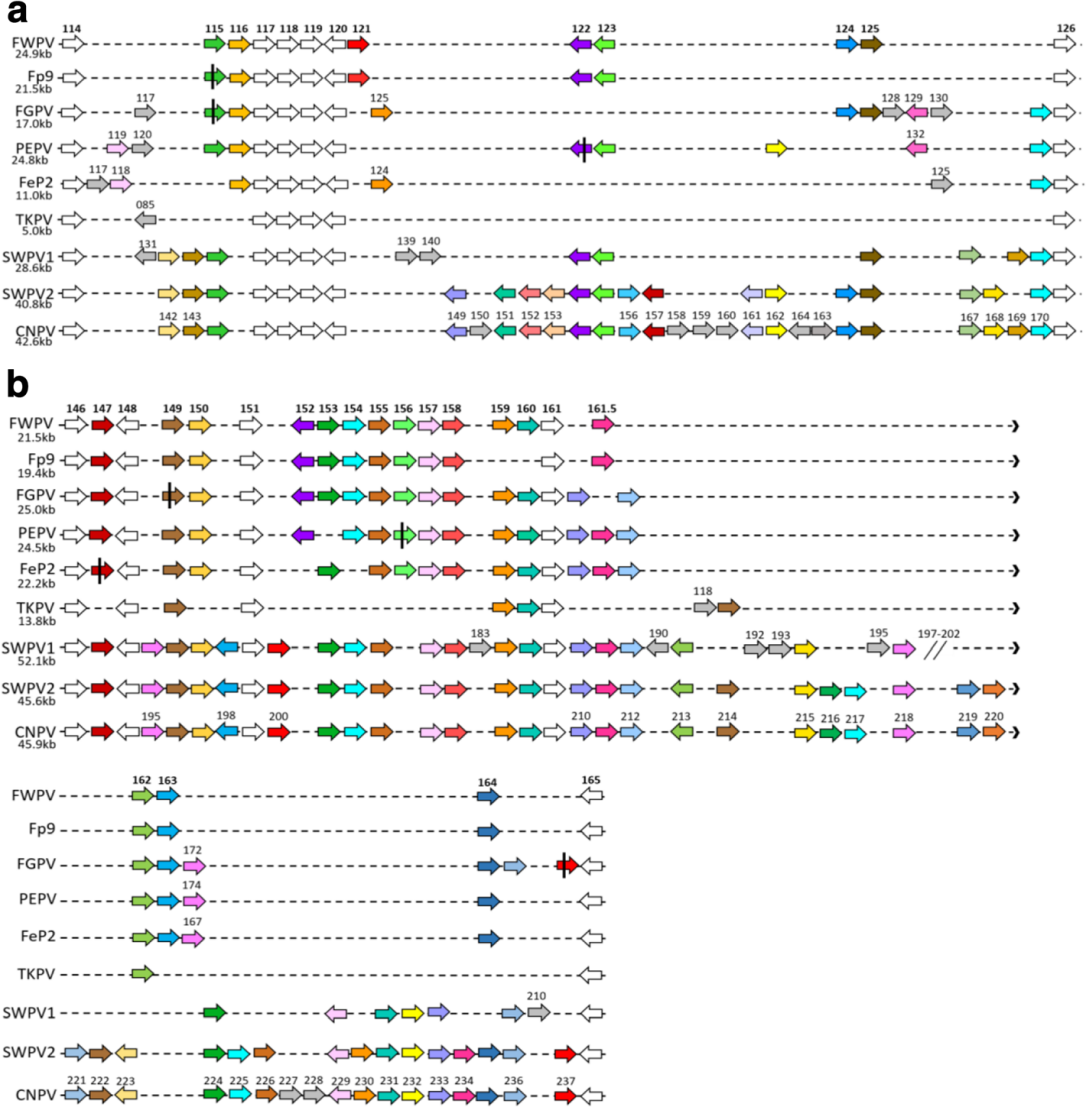
Schematic representing the ORFs present in the rearranged regions **a**) fwpv114–126 and **b**) fwpv146–165 in nine avian poxvirus genomes. ORFs are represented as arrows pointing in the direction of transcription. Numbers below the virus labels show the length of each region in kilobase pairs. (White = present in all genomes; Grey = unique to one genome; Coloured = present in 2–6 genomes or present in all genomes but with one or more orthologues not intact. Homologous, syntenic ORFs are shaded in the same colour across genomes (some colours have been repeated across the length of the genomes but do not indicate synteny or homology – only ORFs of the same colour (excluding grey) and directly above or below each other are syntenic homologues); Black vertical bar = fragmented and/or truncated ORF). Alignment is not to scale and ORF colours do not correspond between figures

Dotplots were created comparing FGPV with other sequenced avian poxvirus genomes and VACV (Copenhagen) to compare overall genomic synteny. This analysis showed the FGPV genome to be highly syntenic with FWPV, PEPV and FeP2 genomes overall and to show major differences compared to CNPV, TKPV, and the two, SWPV genomes, as expected, due to the large differences in genome size. Also notable are the two large breaks in synteny located in the central regions of the dotplots, indicated by arrows (Fig. 2). As was seen in the FeP2 and TKPV genomes [10, 11], a large, rearranged region is present in the FGPV genome between fgpv116–132 (fwpv114–126; cnpv141–171 – green arrows), shown as an alignment schematic in fig. 3a. A second area of rearrangement is also noted between fgpv152–178 (fwpv146–165; cnpv192–238 – red arrows), shown as an alignment schematic in fig. 3b. Regions of rearrangement are referred to from here onward by the FWPV gene annotations as it is the prototype of the genus.

**Fig. 4:**
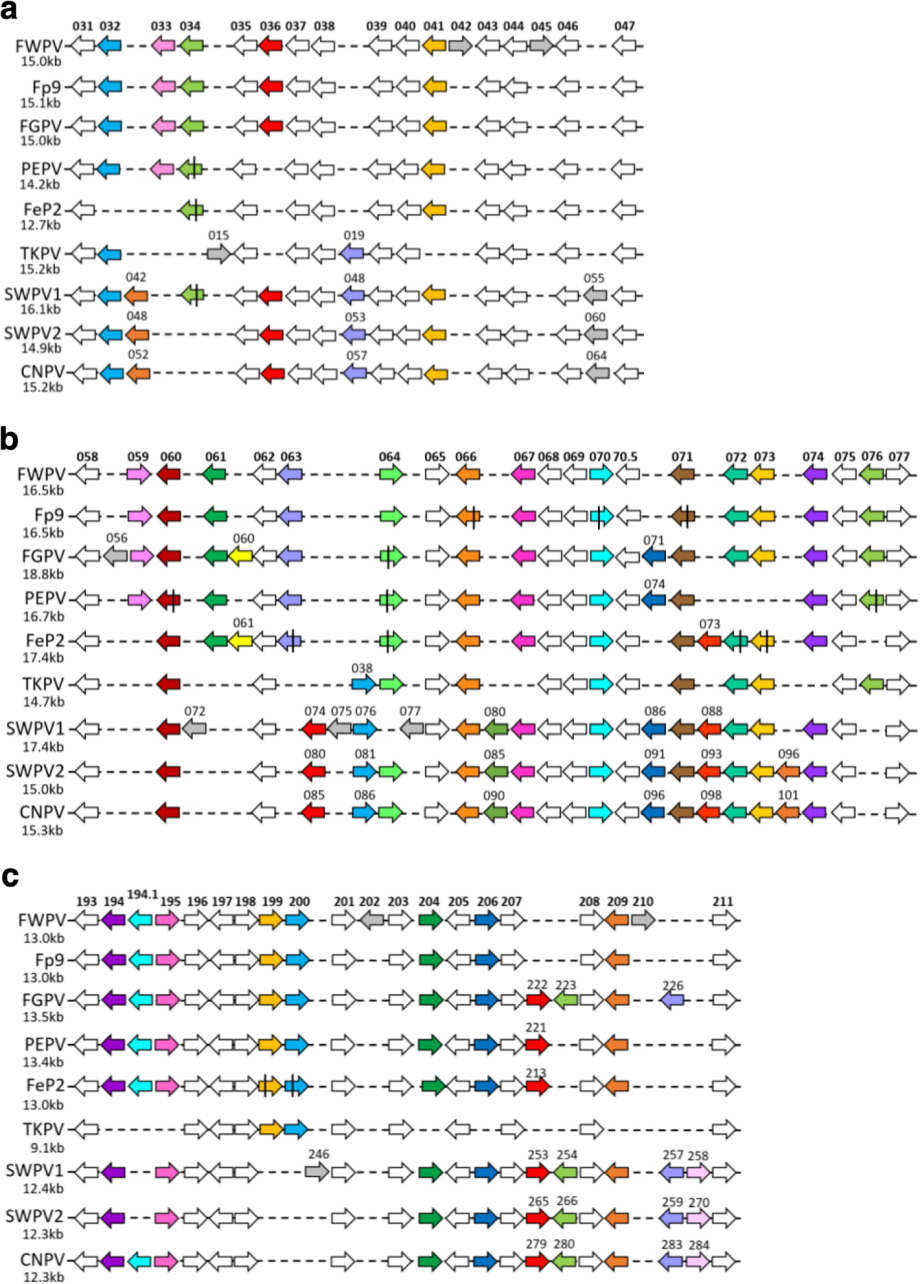
Schematic representing the ORFs present in the rearranged regions a) fwpv031–047, b) fwpv058–077 and c) fwpv193–211 in nine avian poxvirus genomes. Annotations are depicted as in Fig. 3

**Fig. 5:**
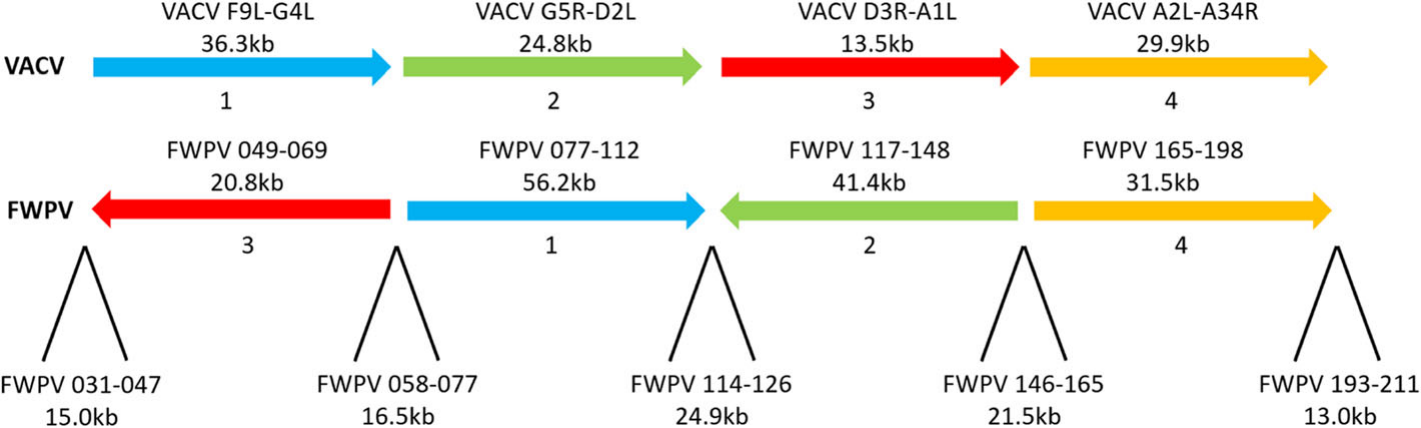
Genome schematic of VACV and FWPV genomes showing four regions conserved in gene content and synteny and five regions of difference. The four conserved regions are annotated as in Gubser et al., 2004 and are not drawn to scale

Analysis of full genome alignments revealed three more regions of difference closer to the boundaries of the cores and the termini. These regions fwpv031–047 (fgpv030–044; cnpv050–065), fwpv058–077 (fgpv055–078; cnpv082–104) and fwpv193–211 (fgpv207–227; cnpv267–285) (Fig. 4a–c) were more similar in size across genomes and therefore not as easily visible in the dotplots. Figure 2H) shows a dotplot comparison of FGPV and VACV (strain Copenhagen). Regions of identity have been highlighted as coloured lines in the inset image which correspond to the conserved regions depicted in Fig. 5.

Figure 5 shows a genome schematic of VACV compared to FWPV with each of the four conserved segments previously identified [21] in different colours as in Fig. 2h). The regions of difference identified in this study are shown below these segments, with ORF numbers and size in kilobases for FWPV. The regions of difference largely correspond with the definitions of the conserved regions identified previously although some overlap is present.

### Core/conserved ORFs

Ninety ORFs have been noted to be conserved in all ChPV genomes [21, 34, 35]. VACV F16 L was erroneously added to the above list, as this ORF is not present in avian poxvirus genomes; and two ORFs (fwpv194 and fwpv194.1) and their equivalents in the other genomes, previously considered conserved among ChPV, have been excluded from the list as they are not present in the TKPV genome. A fourth ORF, fwpv103 was also removed as it is truncated/fragmented in the TKPV genome (tkpv074). ORF fwpv095 and the relative equivalents were also removed as no orthologue is present in SWPV2. Lastly, orthologues fwpv168 (288aa) and cnpv241 (215aa) differ in length by 25% and were excluded. Therefore, in this study, 83 ORFs are considered to be conserved amongst ChPV. It was also previously, noted that a further 89 ORFs were conserved between FWPV, CNPV, FeP2 and PEPV [10]. This list has been updated with the addition of the four, more recent genomes (TKPV, SWPV-1, SWPV-2 and FGPV) and exclusion of ORFs that differ in length by more than 20% bringing the total to 47 (Table 3) and bringing the total number of conserved ORFs in the sequenced avian poxvirus genomes to 130. The TKPV genome is considerably smaller than the other avian poxvirus genomes and as such, is the only genome missing an ORF that would otherwise be conserved in 32 cases (data not shown). Of these 32 ORFs, 28 are either hypothetical proteins or members of gene families suggesting that they are unlikely to be essential to the viral life cycle. Several ORFs were identified that were unreported in the study describing the TKPV genome [11]. Eight of these (tkpv63.1, tkpv86.1, tkpv121.1, tkpv127.1 and tkpv130.1–130.4) are of the 83 ORFs considered to be conserved in all ChPV genomes. A further two ORFs (tkpv1.1 and tkpv151.1) in common amongst avian poxviruses and ORF tkpv60.1, which is not conserved, were also identified on further inspection.

**Table 3:** 47 ORFs found to be uniquely conserved in each of the fully sequenced avian poxvirus genomes

FWPV	CNPV	PEPV	FeP2	FGPV	TKPV	SWPV1	SWPV2	Function
016	032	019	019	011	001.1a	024	028	Ig-like domain
017	033	020	020	012	002	025	029	V-type Ig domain
020	038	024	024	017	005	028	034	C4L/C10L protein
021	039	025	025	018	006	029	035	GPCR
022	040	026	026	019	007	030	036	Ankyrin repeat
023	041	027	027	020	008	031	037	Ankyrin repeat
024	042	028	028	021	009	032	038	Ankyrin repeat
030	048	035	035	029	012	038	044	Alkaline phosphodiesterase
031	050	036	036	030	013	040	046	Ankyrin repeat
035	053	040	038	034	016	044	049	Hypothetical protein
037	055	041	039	036	017	046	051	Hypothetical protein
039	058	043	041	038	020	049	054	B-cell lymphoma 2 (Bcl-2)
040	059	044	042	039	021	050	055	Serpin
043	061	046	044	041	022	052	057	DNA ligase
044	062	047	045	042	023	053	058	Serpin family
046	063	048	046	043	024	054	059	Hydroxysteroid dehydrogenase
047	065	049	047	044	025	056	061	Semaphorin
048	068	050	048	045	026	059	064	GNS1/SUR4
054	076	056	054	051	032	066	072	mutT motif
065	088	067	065	064	040	078	083	Hypothetical protein
068	092	070	068	067	042	082	087	Hypothetical protein
070	094	072	070	069	044	084	089	T10-like protein
071	097	075	072	072	046	087	092	Hypothetical protein
075	103	078	077	076	050	092	098	N1R/p28
086	113	089	087	087	060	102	108	Thymidine kinase
091	118	095	093	093	065	107	113	Hypothetical protein
092	119	096	094	094	066	108	114	Hypothetical virion core protein
104	131	108	106	106	075	120	126	Hypothetical protein
105	132	109	107	107	076	121	127	Hypothetical protein
110	137	114	112	112	080	126	132	Hypothetical protein
113	140	117	115	115	083	129	135	Hypothetical protein
145	191	153	146	151	109	167	179	Hypothetical protein
151	199	159	153	157	113	175	187	Deoxycytidine kinase
190	264	203	195	204	140	237	250	A-type inclusion protein
191	265	204	196	205	141	238	251	A-type inclusion protein
196	270	210	202	211	144	243	256	Hypothetical protein
201	273	215	207	216	149	247	259	Hypothetical protein
203	274	216	208	217	150	248	260	Tyrosine kinase
205	276	218	210	219	151	250	262	Hypothetical protein
207	278	220	212	221	151.1a	252	264	Hypothetical protein
208	281	222	214	224	152	255	267	Hypothetical protein
211	285	225	216	227	153	259	271	Epidermal Growth Factor
212	286	226	217	228	154	260	272	Serine/threonine protein kinase
213	287	227	218	229	155	261	273	Hypothetical protein
214	289	228	219	230	156	263	275	Putative 13.7 kDa protein
219	296	234	226	238	161	272	282	Ankyrin repeat
232	304	248	238	251	164	283	290	Ankyrin repeat

^a^tkpv 001.1 and 151.1 as well as ^a^swpv1 241.1 and ^a^swpv2 254.1 were not reported in the literature [11, 12], but were identified on inspection of the sequences deposited in Genbank

A concatenated nucleotide alignment of the 130 conserved ORFs from each of the sequenced virus genomes shows FGPV to have the greatest degree of identity to the other South African isolates, PEPV and FeP2 (~96%) followed by FWPV (~90%), the clade B isolates (68–70%) and lastly TKPV (~65%) (Table 4). An amino acid alignment showed percentage identities to be very similar with a maximum difference of 3% compared to the equivalent nucleotide identity (data not shown).

**Table 4:** Pairwise comparison of the % identity and number of differences between nucleotide alignments of 130 conserved genes in eight avian poxvirus genomes

	FWPV	FeP2	PEPV	FGPV	CNPV	SWPV1	SWPV2	TKPV
FWPV		12,486	12,550	11,763	40,283	39,648	40,454	44,591
FeP2	90.2		4967	4636	39,532	39,000	39,705	44,412
PEPV	90.2	96.1		4548	39,558	39,041	39,733	44,468
FGPV	90.8	96.4	96.5		39,719	39,138	39,893	44,602
CNPV	68.8	69.4	69.4	69.3		27,136	1228	45,260
SWPV1	69.3	69.8	69.8	69.7	78.9		27,405	44,385
SWPV2	68.7	69.3	69.3	69.2	99.0	78.7		45,466
TKPV	65.6	65.8	65.8	65.8	65.2	65.8	65.0	

% identities are shown in the lower left and the number of nucleotide differences shown in the upper right

### Multigene families

Avian poxviruses contain several, large, multigene families with immune related functions that can make up close to 50% of the genome. Table 5 below outlines the copy numbers of each of the 14 multigene families identified in the FGPV genome compared to that of the other sequenced avian poxvirus genomes. Overall FGPV has a similar complement of multi-gene families but has significantly more ankyrin repeat family genes than are found in other clade A viruses.

**Table 5:** Copy number of ORFs in each of the 14 multi-gene families identified in each of the fully sequenced avian poxvirus genomes

Gene family	FWPV	FP9	CNPV	PEPV	FeP2	TKPV	FGPV	SWPV1	SWPV2
Ankyrin Repeat	31	22	51	33	26	16	45	50	46
B22R	6	5	6	5	4	1	4	6	7
N1R/p28	10	8	26	11	11	3	13	20	20
C4L/C10L	3	3	3	2	2	2	2	2	3
CC chemokine	4	4	5	1	4	2	6	6	5
C-type lectin	9	6	11	7	4	2	4	13	11
GPCR	3	2	4	3	2	2	3	4	4
HT motif	6	6	5	5	4	1	7	4	4
Ig-like domain	5	4	9	6	4	3	9	9	8
Serpin	5	5	5	4	4	3	5	5	5
EFc	3	2	2	1	1	1	1	2	2
TGF-β	1	1	5	1	1	1	1	3	4
Β-NGF	2	2	2	0	0	2	3	2	2
IL-18 BP	1	1	3	1	0	2	0	3	3
TOTAL	89	71	137	80	67	41	103	129	124
% of TOTAL ORFs	34	29	42	28	25	24	36	42	40

### Reticuloendotheliosis virus (REV)

REV insertions are typically found between fwpv201–203. The entire FGPV genome was searched for the presence of REV LTRs as well as the *gag*, *pol* and *env* genes. None of the REV elements were found anywhere in the FGPV genome.

## Discussion

Overall, the FGPV genome was found to be similar to other avian poxvirus genomes in terms of genome size, AT content and number of ORFs but was also found to be distinct from these genomes in several ways. FGPV was found to have a unique complement of gene family proteins as well as several ORFs that were truncated, fragmented or extended relative to their closest orthologues. The majority of these altered ORFs were found in the terminal regions and encode hypothetical or gene family proteins which is expected as they are largely involved in virus-host interactions which are host specific. Also present in the terminal regions are the majority of the genes of interest that are only present in a subset of avian poxvirus genomes, if at all. Three FGPV ORFs were discovered (fgpv 117, 232 and 256) that have yet to be identified in other avian poxvirus genomes and the FGPV genome differed from all other avian poxvirus genomes at the regions of difference identified in this analysis. A nucleotide alignment of 130 conserved ORFs showed FGPV to be most closely related to South African isolates PEPV and FeP2, followed by FWPV, the clade B isolates and lastly TKPV.

This paper confirms the differences between avipoxviruses and orthopoxviruses in gene synteny. The FWPV genome was first shown to exhibit major organisational differences compared to the genome of Vaccinia virus (VACV) using restriction enzyme mapping. It was shown that large segments of the FWPV genome had been reversed and/or translocated relative to VACV although gene content appeared to be largely maintained [36]. Sequencing of the FWPV genome and other ChPV genomes allowed for more detailed comparisons which showed that the core region forms a continuous block in all ChPVs except parapoxviruses and avian poxviruses due to various genome rearrangements. It was specifically noted that the core region of avian poxviruses has broken into four segments two of which have been reversed and one of which has been translocated [7, 21]. Sequencing of the CNPV genome allowed comparison of the regions found between these four segments, to the equivalent FWPV regions, and showed major differences in gene content. At the time, it was unclear if these differences were due to subclade specificities or were a feature of all avian poxviruses.

Although the overall genome architecture of avian poxviruses is largely conserved, with the expected variability in the termini, a pattern is emerging with all sequenced isolates exhibiting major differences in multiple, defined, central regions. FP9 was the first clade A isolate noted to be somewhat different to FWPV in the fwpv114–126 region with the truncation of fp9 115 and deletion of fp9 125 and fp9 126 [8]. FeP2 was then noted to have a large deletion of over 10 kb and although this region in the PEPV genome was of similar length to FWPV, several inserted and deleted ORFs were noted [10]. In the TKPV genome, ORF tkpv085 (fwpv114) was identified as being affected by genomic rearrangement [11]. In this study, alignment of this region in all clade A viruses shows a large variation in length from ~11 kb in Fep2, to over 24 kb in FWPV and PEPV. In FGPV this region spans 16.5 kb and encodes 16 ORFs (Fig. 3a). If this comparison is expanded to avian poxviruses in other clades, the difference is much larger with a variation in length from ~5 kb (encoding seven ORFs) in TKPV to over 42.6 kb (encoding 31 ORFs) in CNPV. Four ORFs in this region (fwpv117 - fwpv120 and the relative equivalents) are conserved among all viruses and syntenic as would be expected, as they are of the 83 genes conserved and considered essential among all ChPVs. What is unexpected is the placement and retention of this pocket of four essential genes in a region of highly divergent gene content and synteny. ORF fwpv117 encodes a putative nuclease involved in viral DNA replication [37], fwpv118 encodes RNA polymerase subunit RPO7 fwpv119 is of unknown function and fwpv120 encodes a virion core protein involved in several stages of virion morphogenesis [38]. This region also contains several ORFs unique to avian poxviruses.

The second region of difference found in the genome core shows less difference in length among clade A viruses from ~19 kb in Fp9 to ~25 kb in FGPV but as above, when including viruses in other clades this difference in length increases considerably from ~14 kb in TKPV (encoding 17 ORFs) to ~52 kb in SWPV1 (encoding 42 ORFs) (Fig. 3b). Only one ORF (fwpv148) conserved in all ChPV is present in this region and encodes a virion protein involved in immature virion formation [39].

The three regions of difference found closer to the termini (Fig. 4a), b) and c)) are more similar in size across genomes and contain several more conserved ORFs. Again, this is unusual for poxviruses as we would expect these regions to be less conserved compared to the two, central regions of difference. The three clade B viruses (CNPV, SWPV1 and SWPV2) are more similar to each other in terms of gene content at these locations than the clade A viruses and CNPV and SWPV2 are nearly identical as expected based on the conserved ORF identities and phylogenetic analysis.

Poxviruses have been known to use gene duplication and subsequent, mutationally driven, diversification of paralogues to their advantage to combat host immune responses. Elde et al., 2012, specifically looked at the ability of VACV to adapt to growth in human cells where the host range factor K3 L is non-functional. E3L functions similarly to K3 L and is functional in human cells. It was found that when E3L was deleted, leaving the virus susceptible to host antiviral responses, the K3 L gene was recurrently amplified, with each of the paralogues able to explore mutational space until an adaptive substitution was found. Effective copies of the K3 L gene were retained and the others lost over generations. It was also noted that duplications other than K3 L all occurred in the terminal regions of the VACV genome [40].

In the case of avian poxviruses, it is interesting to note that several ORFs in the fwpv114–126; cnpv141–171 region of difference of CNPV, SWPV1 and SWPV2 are present as repeats/paralogues of gene family proteins: cnpv143–144 = ANK repeat; cnpv150–151 = ANK repeat; cnpv154–155 = B22R; cnpv157–158 = TGF-β; cnpv159–160 = N1R/p28; cnpv161–162 = TGF-β; cnpv166–167 = Ig-like domain; cnpv168–169 = N1R/p28.In the second region of difference (fwpv146–165; cnpv192–238)) found in the central region, the CNPV genome has 18 copies of N1R/p28 like proteins in this region while SWPV1 and SWPV2 contain differing subsets of these, which may be the result of genomic accordions at work earlier in their evolutionary histories.

## Conclusions

Genome sequencing and comparative genomics are the gold standards in terms of determining phylogenetic and evolutionary relationships among viral species and explaining differences in host range and pathogenicity. Several important bird species and commercial flocks have been shown to be severely affected by avian poxvirus infection. This study provides the genome sequence of a novel, South African isolate from lesser flamingos and provides insight into overall genome architecture that appears to be unique to avian poxviruses. Given the relative conservation of the central region of other poxvirus genomes, the regions of difference identified here are particular areas of interest in avian poxvirus genomics, but it is currently unclear why these regions would be so susceptible to rearrangement. The mechanisms responsible for such large-scale rearrangements are also yet to be elucidated. As more avian poxvirus genomes are sequenced, exploration and confirmation of these intriguing differences in these important pathogens can be conducted.

## Additional files

Additional file 1: FGPV Open Reading Frames. Location and function of FGPV open reading frames and comparison to their closest orthologues (PDF 756 kb)
