# Supplementary material for: Comparative analysis of avian poxvirus genomes, including a novel poxvirus from lesser flamingos (Phoenicopterus minor), highlights the lack of conservation of the central region
Source: BMC Genomics. 2017 Dec 6;18:947. doi: 10.1186/s12864-017-4315-0 (PMC5718139; doi:10.1186/s12864-017-4315-0)
Supplement: Supplementary file 1 — FGPV Open Reading Frames. Location and function of FGPV open reading frames and comparison to their closest orthologues (PDF 756 kb) [file 12864_2017_4315_MOESM1_ESM.pdf]

Additional file 1: FGPV Open Reading Frames

ORF	ORF length (aa)	Status (I, F, T, E)*	Closest Homologues (HOM)	HOM length (aa)	FGPV vs HOM BLASTx Identity (%)	VACV HOM	Description / Putative function
**001**	474	I	cnpv 017/tkpv009/fpv034/fep226	486	52/28/29/29	B4R	Ankyrin repeat family
**002**	682	I	fep010/pepv010/cnpv009/fpv244/tkpv009	680	95/96/32/32/28	B4R	Ankyrin repeat family
**003**	355	I	fep011/pepv011/fpv010/cnpv022	355	95/96/86/65	C12L	Serpin family
**004**	293	I	pepv012/fep012/fpv011/cnpv025	288	95/94/81/58	-	α-SNAP
**005**	520	I	pepv013/fep013/cnpv026/fpv246	518	89/89/53/31	B4R	Ankyrin repeat family
**006**	174	I	pepv014/fep014/*Ficedula albicollis*/cnpv018	174	88/86/37/30	-	Interleukin 10
**007**	330	I	pepv015/fep015/fpv012/cnpv030	329	91/88/80/46	K1L	Ankyrin repeat family
**008**	402	I	pepv016/fep016/cnpv028/fpv240	402	93/92/40/31	B4R	Ankyrin repeat family
**009**	437	I	pepv017/fep017/fpv014/cnpv019/tkpv166	437	95/95/87/50/39	B4R	Ankyrin repeat family
**010**	171	I	pepv018/fep018/fpv015/cnpv013	171	90/88/78/40	-	Hypothetical protein
**011**	239	I	fep019/pepv019/fpv016/cnpv032	237	92/89/84/32	-	Ig-like domain (putative IFN gamma BP)
**012**	245	I	fep020/pepv020/fpv017/tkpv002/cnpv033	245	91/90/78/40/41	-	V-type Ig domain
**013**	684	I	fep021/pepv021/fpv018/cnpv034/tkpv166	683	92/92/85/43/25	C9L	Ankyrin repeat family
**014**	113	I	pepv022/fep022/fpv019/cnpv036	113	92/91/73/29	-	Hypothetical protein
**015**<br>**016**	100<br>47	T<br>F	fep023/pepv023/cnpv037	189	-	-	Hypothetical protein
**017**	428	I	fep024/pepv024/fpv020/cnpv038/tkpv119	427	95/93/87/43/34	C10L	C4L/C10L protein
**018**	337	I	pepv025/fep025/fpv021/cnpv039/tkpv006	334	95/92/81/46/37	-	G protein-coupled receptor family
**019**	580	I	fep026/pepv026/fpv022/cnpv040/tkpv007	581	95/91/88/37/31	B4R	Ankyrin repeat family
**020**	434	I	fep027/pepv027/fpv023/cnpv041/tkpv008	434	97/96/91/58/49	M1L	Ankyrin repeat family
**021**	595	I	fep028/pepv028/fpv024/cnpv042/tkpv009	594	97/96/91/50/47	B4R	Ankyrin repeat family
**022**	203	I	pepv029/fep029/fpv025/cnpv043	203	99/95/90/55	-	Hypothetical protein
**023**	498	I	fep030/pepv030/cnpv044/fpv024/tkpv009	498	92/93/43/34/34	B4R	Ankyrin repeat family
**024**	406	I	pepv031/fpv026/tkpv010/cnpv151/fep031	406	94/85/40/30/29	B4R	Ankyrin repeat family
**025**	333	I	pepv032/fpv027/cnpv045/tkpv011	332	94/86/43/44	-	G-protein-coupled receptor family
**026**	180	I	pepv033/fpv028	180	95/89	-	Hypothetical protein
**027**	464	I	fep033/cnpv046/tkpv163/fpv232/pepv252	464	96/51/32/28/29	-	Ankyrin repeat family
**028**	126	I	pepv034/fep034/fpv029/cnpv047	126	98/96/93/53	-	Peptidyl-tRNA hydrolase
**029**	815	I	pepv035/fep035/fpv030/cnpv048/tkpv012	815	94/93/85/59/42	-	Alkaline phosphodiesterase
**030**	345	I	fep036/fpv031/pepv036/cnpv050/tkpv013	341	96/95/95/44/38	B4R	Ankyrin repeat family
**031**	382	I	pepv037/fpv032/cnpv051/tkpv014	375	91/88/50/43	-	DNase II
**032**	291	I	pepv038/fpv033	291	95/89	-	α-SNAP
**033**	411	I	fpv034/cnpv017 (fep and pepv fragments)	415	84/35	B4R	Ankyrin repeat family
**034**	135	I	pepv040/fpv035/fep038/cnpv053/tkpv016	135	97/96/96/51/51	-	Hypothetical protein
**035**	131	I	fpv036/cnpv054	153	79/35	-	Hypothetical protein
**036**	164	I	fep039/pepv041/fpv037/tkpv017/cnpv055	163	88/87/70/39/42	-	Hypothetical protein
**037**	145	I	fep040/pepv042/fpv038/cnpv056	145	97/97/93/71	F2L	dUTP pyrophosphatase
**038**	175	I	fep041/pepv043/fpv039/cnpv058/tkpv020	175	94/94/83/40/40	-	B-cell lymphoma 2 (Bcl-2)
**039**	337	I	fep042/pepv044/fpv040/tkpv021/cnpv059	337	97/96/92/49/51	C12L	Serpin family

**040**	212	I	pepv045/fep043/fpv041/cnpv060	220	89/89/67/24	-	Hypothetical protein
**041**	564	I	fep044/pepv046/fpv043/cnpv061/tkpv022	564	98/98/91/70/64	A50R	DNA ligase
**042**	358	I	fep045/pepv047/fpv044/cnpv062/tkpv023	358	98/97/92/48/49	C12L	Serpin family
**043**	368	I	fep046/pepv048/fpv046/cnpv063/tkpv024	370	96/95/83/62/56	A44L	Hydroxysteroid dehydrogenase
**044**	576	I	fep047/pepv049/fpv047/cnpv065/tkpv025	612	95/95/80/52/46	A39R	Semaphorin
**045**	261	I	pepv050/fep048/fpv048/cnpv068/tkpv026	261	99/98/95/83/71	-	GNS1/SUR4
**046**	154	I	pepv051/fpv049/fep049/tkpv027/cnpv069	154	97/95/96/75/71	A1L	Late transcription factor VLTF-2
**047**	552	I	fep050/pepv052/fpv050/tkpv028/cnpv070	552	99/99/97/82/82	D13L	Rifampicin resistance, N3L protein
**048**	289	I	fep051/pepv053/fpv051/cnpv071/tkpv029	289	99/99/96/76/77	D12L	mRNA capping enzyme, small subunit
**049**	637	I	fep052/fpv052/pepv054/cnpv074/tkpv030	637	99/96/99/83/84	D11L	NPH-1 transcription termination factor
**050**	225	I	pepv055/fep053/fpv053/tkpv031/cnpv075	225	98/98/96/70/67	D10R	mutT motif
**051**	237	I	fep054/pepv056/fpv054/cnpv076/tkpv032	237	97/97/96/77/73	D9R	mutT motif
**052**	274	I	pepv057/fep055/fpv055/cnpv166	274	93/92/66/41	-	V-type Ig domain
**053**	161	I	fep056/pepv058/fpv056/tkpv033/cnpv078	161	99/98/97/76/75	D7R	RNA polymerase subunit RPO18
**054**	633	I	fep057/pepv059/fpv057/cnpv080/tkpv034	633	99/99/99/94/92	D6R	Early transcription factor small subunit, VETFS
**055**	791	I	pepv060/fep058/fpv058/tkpv035/cnpv082	791	99/99/98/84/80	D5R	NTPase; DNA replication
**056**	251	F	cnpv072	312	36	-	CC chemokine family
**057**	219	I	pepv061/fpv059	219	92/90	-	Deoxycytidine kinase
**058**	196	I	fep059/fpv060	200	90/84	-	CC chemokine family
**059**	128	I	fep060/fpv061/pepv063/tkpv059	109	93/76/82/61	-	CC chemokine family
**060**	196	I	fep061	199	90	-	CC chemokine family
**061**	218	I	fpv062/pepv064/fep062/cnpv084/tkpv037	218	99/98/98/83/82	D4R	Uracil DNA glycosylase
**062**	378	I	pepv065/fpv063/cnpv216	401	94/81/38	-	Hypothetical protein
**063**	134	F	fpv064/cnpv087/tkpv039	200	94/78/74	-	Glutathione peroxidase
**064**	110	I	fep065/fpv065/pepv067/tkpv040/cnpv088	110	97/95/81/38/37	-	Hypothetical protein
**065**	137	I	pepv068/fep066/fpv066/cnpv089/tkpv041	137	95/95/84/54/51	-	Hypothetical protein
**066**	85	I	fep067/pepv069/fpv067/cnpv091	93	100/99/96/63	-	HT motif family
**067**	130	I	fep068/pepv070/fpv068/tkpv042/cnpv092	131	88/84/66/61/59	-	Hypothetical protein
**068**	269	I	pepv071/fep069/fpv069/cnpv093/tkpv043	269	97/97/95/54/54	D3R	Virion protein
**069**	273	I	pepv072/fep070/fpv070/cnpv094	273	97/95/87/54	-	T10-like protein
**070**	43	I	fep071/pepv073/fpv70.5/cnpv095	43	100/93	-	Hypothetical protein
**071**	77	I	Ubiquitin 40S ribosomal protein	110	100	-	Ubiquitin family
**072**	287	I	pepv075/fep072/fpv071/tkpv046/cnpv097	287	94/93/89/51/51	-	Hypothetical protein
**073**	186	I	fpv072/cnpv099/tkpv047	186	88/57/58	-	β-NGF-like family
**074**	176	I	fpv073/cnpv100/tkpv048	174	75/55/38	-	IL-18 binding protein
**075**	105	I	pepv077/fep076/fpv074	104	94/95/83	-	Hypothetical protein
**076**	187	I	pepv078/fep077/fpv075/cnpv103/tkpv050	211	97/96/83/61/41	-	N1R/p28 family
**077**	147	I	fpv076/cnpv279	144	67/33	-	β-NGF-like family
**078**	125	I	pepv080/fpv077/tkpv051/cnpv104	125	100/95/64/61	G4L	Glutaredoxin
**079**	225	I	fep079/pepv081/fpv079/cnpv106/tkpv052	225	98/98/96/63/56	G2R	Putative transcriptional elongation factor
**080**	104	I	pepv082/fep080/fpv078/cnpv105/tkpv053	103	99/97/92/65/51	G3L	Hypothetical protein
**081**	238	F	pepv083/fep081/fpv080	336	91/90/73		Transforming Growth Factor (TGF-β)

**082**	626	I	pepv084/fep082/fpv081/cnpv108/tkpv054	624	98/97/95/71/67	G1L	Metalloprotease / G1 glycoprotein
**083**	682	I	pepv085/fep083/fpv082/cnpv109/tkpv055	682	98/98/95/75/73	I8R	DNA/RNA helicase/NPH-11
**084**	421	I	fep084/pepv086/fpv083/cnpv110/tkpv056	421	99/98/97/74/70	I7L	Virion core peptidase
**085**	390	I	pepv087/fep085/fpv084/cnpv111/tkpv057	390	99/99/95/69/63	I6L	DNA-binding protein
**086**	81	I	pepv088/fpv085/cnpv112/tkpv058	81	100/90/74/80	I5L	IMV membrane protein
**087**	185	I	pepv089/fep087/fpv086/cnpv113/tkpv060	183	95/94/88/65/61	J2R	Thymidine kinase
**088**	91	I	pepv090/fep088/fpv087/cnpv114/tkpv062	91	96/93/92/56/54	-	HT motif family
**089**	291	I	fep089/fpv088/pepv091/tkpv063/cnpv115	291	96/95/95/60/61	I3L	ssDNA binding phosphoprotein
**090**	65	I	fpv089/pepv092/fep090/cnpv116	65	98/97/95/74	I2L	Hypothetical protein
**091**	311	I	pepv093/fep091/fpv090/cnpv117/tkpv064	311	99/99/98/85/86	I1L	DNA binding virion core protein
**092**	34	I	fpO3L/pepv094/fep092	34	94/97/97	O3L	MV entry/fusion complex
**093**	656	I	pepv095/fep093/fpv091/cnpv118/tkpv065	656	97/97/90/55/49	O1L	Hypothetical protein
**094**	131	I	pepv096/fpv092/fep094/cnpv119/tkpv066	131	98/96/97/64/56	E11L	Hypothetical virion core protein
**095**	130	3’ E	pepv097/fep095/fpv093/cnpv120/tkpv067	94	100/96/92/78/82	E10R	Sulfhydryl oxidase
**096**	989	I	fpv094/pepv098/fep096/cnpv121/tkpv068	988	96/96/96/78/75	E9L	DNA polymerase
**097**	282	I	fep097/pepv099/fpv095/tkpv069/cnpv122	282	99/97/93/66/63	E8R	Hypothetical protein
**098**	571	I	pepv100/fep098/fpv096/cnpv123/tkpv070	571	99/99/97/77/74	E6R	Hypothetical protein
**099**	1893	I	pepv101/fep099/fpv097/cnpv124	1894	94/94/85/78	-	VARV B22R family
**100**	1802	I	pepv102/fpv098/fep100/cnpv125	1812	96/88/97/75	-	VARV B22R family
**101**	1935	I	pepv103/fep101/fpv099/cnpv126	1922	94/94/85/59	-	VARV B22R family
**102**	182	I	pepv104/fep102/fpv100/cnpv127/tkpv071	182	99/99/98/87/77	E4L	RNA pol subunit RPO30
**103**	717	I	pepv105/fep103/fpv101/cnpv128/tkpv072	717	98/96/94/61/52	E2L	Hypothetical protein
**104**	472	I	fep104/pepv106/fpv102/cnpv129/tkpv073	472	99/99/98/75/74	E1L	Poly(A) polymerase large subunit, PAP-L
**105**	114	I	fpv103/cnpv130	114	100/71	F17R	DNA binding virion core phosphoprotein
**106**	210	I	fep106/pepv108/fpv104/cnpv131/tkpv075	210	95/95/81/39/41	-	Hypothetical protein
**107**	144	I	pepv109/fep107/fpv105/tkpv076/cnpv132	149	97/97/94/74/73	F15L	Hypothetical protein
**108**	99	I	fep108/pepv110/cnpv133/fpv106/tkpv077	99	96/93/68/77/46	-	Hypothetical protein
**109**	1780	I	pepv111/fep109/fpv107/cnpv134	1779	97/97/86/58	-	VARV B22R family
**110**	376	I	fep110/pepv112/fpv108/cnpv135/tkpv078	377	98/96/94/84/75	F13L	Virion envelope protein
**111**	639	I	pepv113/fep111/fpv109/cnpv136/tkpv079	639	96/96/87/50/45	F12L	Virion release protein
**112**	453	I	pepv114/fpv110/fep112/cnpv137/tkpv080	452	96/85/92/45/44	F11L	Hypothetical protein
**113**	444	I	fep113/pepv115/fpv111/cnpv138/tkpv081	444	99/98/97/76/76	F10L	Serine/threonine protein kinase (virus assembly)
**114**	213	I	pepv116/fep114/fpv112/cnpv139/tkpv082	213	97/97/94/74/70	F9L	Lipid membrane protein of NCLDV
**115**	66	I	fep115/pepv117/fpv113/cnpv140/tkpv083	66	94/97/95/71/62	F8L	Hypothetical protein
**116**	174	I	pepv118/fep116/fpv114/cnpv141/tkpv084	183	97/95/95/77	-	HAL3 domain
**117**	769	I	cnpv223/pepv269/fpv244/tkpv015/fep010	847	28/29/30/27/27	B4R	Ankyrin repeat family
**118**<br>**119**	299<br>171	T<br>F	pepv121/fpv115/cnpv144	542	-	-	Ankyrin repeat family
**120**	123	I	fep119/fpv116	122	94/73	-	CC-chemokine family
**121**	440	I	fep120/pepv123/fpv117/cnpv145/tkpv086	440	98/97/93/66/60	G5R	Flap endonuclease (FEN-1)
**122**	63	I	pepv124/fpv118/fep121/cnpv146	63	100/98/98/74	G5.5R	RNA pol subunit RPO7
**123**	188	I	fep122/pepv125/fpv119/cnpv147/tkpv087	188	99/97/96/69/65	G6R	Hypothetical protein
**124**	343	I	fep123/pepv126/fpv120/cnpv148/tkpv088	343	99/99/97/80/65	G7L	Virion core protein

**125**	174	I	fep124/cnpv012	203	77/33	-	Hypothetical protein
**126**	341	I	cnpv227/fpv124/fep166/pepv174/tkpv115	359	69/70/45/45/44	-	N1R/p28 family
**127**	357	I	cnpv166/fpv125/fep056/pepv057	345	73/61/40/40	B19R	V-type Ig domain
**128**	164	F	cnpv166/fpv125/pepv057/fep056	345	48/49/42/41	B19R	V-type Ig domain
**129**	116	I	pepv132/cnpv086/tkpv038	116	94/50/45	C22L/B28R	TNF receptor like protein
**130**	279	F	cnpv216/fpv063/pepv065	404	54/42/41	-	Hypothetical protein
**131**	217	I	pepv133/fep126/cnpv170	217	97/93/82	A48R	Thymidylate kinase
**132**	260	I	pepv134/fpv126/fep127/cnpv171/tkpv089	260	100/99/99/96/84	G8R	Late transcription factor VLTF-1
**133**	336	I	pepv135/fep128/fpv127/cnpv172/tkpv090	322	99/96/93/73/62	G9R	Myristylated protein
**134**	243	I	pepv136/fep129/fpv128/cnpv173/tkpv091	243	99/99/97/87/84	L1R	Myristylated MV membrane protein
**135**	96	I	pepv137/fep130/fpv129/cnpv174/tkpv092	96	91/91/81/30/	L2R	Hypothetical protein
**136**	297	I	pepv138/fep131/fpv130/cnpv175/tkpv093	301	97/96/92/82/79	L3L	Hypothetical protein
**137**	253	I	fep132/pepv139/fpv131/cnpv176/tkpv094	253	98/97/95/74/67	L4R	DNA binding virion core VP8
**138**	129	I	fep133/pepv140/fpv132/cnpv177/tkpv095	129	98/98/95/77/65	L5R	Putative membrane protein
**139**	148	I	fep134/pepv141/fpv133/cnpv178/tkpv096	148	99/99/97/76/76	J1R	Hypothetical protein
**140**	308	I	pepv142/fep135/fpv134/cnpv179/tkpv097	308	98/97/96/96/70	J3R	PolyA polymerase (PAPs)
**141**	186	I	pepv143/fep136/fpv135/tkpv098/cnpv180	186	98/98/96/76/75	J4R	RNA pol Subunit RPO22
**142**	137	I	fep137/pepv144/fpv136/cnpv181/tkpv099	138	97/96/94/79/75	J5L	Membrane protein
**143**	1287	I	pepv145/fep138/fpv137/cnpv182/tkpv100	1287	99/99/98/89/87	J6R	RNA pol Subunit RPO147
**144**	166	I	fep139/pepv146/fpv138/cnpv183/tkpv101	166	99/98/95/81/80	H1L	Protein tyrosine Phosphatase
**145**	190	I	pepv147/fep140/fpv139/cnpv184/tkpv102	190	100/99/97/87/83	H2R	Hypothetical protein
**146**	333	I	fpv140/pepv148/fep141/cnpv186/tkpv104	327	99/97/96/57/55	H3L	Virion envelope protein (p35)
**147**	799	I	pepv149/fep142/fpv141/cnpv187/tkpv105	799	99/98/97/82/79	H4L	RNA polymerase associated protein RAP94
**148**	172	I	pepv150/fep143/fpv142/cnpv188/tkpv106	174	95/95/91/51/52	H5R	Late transcription factor VLTF-4
**149**	318	I	pepv151/fep144/fpv143/cnpv189/tkpv107	316	99/99/97/81/75	H6R	DNA topoisomerase
**150**	152	I	pepv152/fep145/fpv144/cnpv190/tkpv108	152	97/97/93/53/49	H7R	Putative 17 kDa protein
**151**	103	I	pepv153/fep146/fpv145/cnpv191/tkpv109	103	93/90/90/47/45		Hypothetical protein
**152**	852	I	pepv154/fep147/fpv146/ tkpv110/cnpv192	822	98/98/95/76/73	D1R	mRNA capping enzyme, large subunit
**153**	109	I	pepv155/fpv147/cnpv193	104	96/89/58	-	HT motif family
**154**	140	I	pepv156/fep149/fpv148/cnpv194/tkpv111	140	99/98/90/60/56	D2L	Virion protein
**155**	145	T	pepv157/fep151/fpv149/cnpv196/tkpv112	189	98/98/86/56	-	Hypothetical protein
**156**	284	I	fep152/pepv158/fpv150/cnpv197/tkpv113	284	93/93/85/44/49	-	N1R/p28 family
**157**	238	I	pepv159/fep153/fpv151/cnpv199	238	95/94/86/52	-	Deoxycytidine kinase
**158**	127	I	pepv160/fpv152/cnpv282	127	94/83/36	-	HT motif family
**159**	208	I	fep154/fpv153/cnpv201	209	93/79/55	-	Hypothetical protein
**160**	276	I	pepv161/fpv154/cnpv202	270	93/80/31	-	N1R/p28 family
**161**	411	I	fep155/pepv162/fpv155/cnpv203	408	94/93/88/36	-	N1R/p28 family
**162**	136	I	fep156/fpv156	132	96/86	-	HT motif family
**163**	328	I	fep157/pepv164/fpv157/cnpv205	327	94/94/85/48	-	N1R/p28 family
**164**	464	I	fep158/pepv165/fpv158/cnpv206	464	98/97/95/74	-	Photolyase
**165**	246	I	fep159/pepv166/fpv159/cnpv207/tkpv121	246	97/96/85/46/40	-	N1R/p28 family
**166**	156	I	pepv167/fep160/fpv160/cnpv208/tkpv116	156	99/98/95/42/36	-	Hypothetical protein
**167**	149	I	fep161/pepv168/fpv161/cnpv209/tkpv117	149	98/97/92/48/43	-	N1R/p28 family

**168**	133	I	pepv169/fep162/cnpv210/fpv124	133	98/95/45/40	-	N1R/p28 family
**169**	160	I	pepv171/fep164/cnpv212/fpv124	160	90/89/58/44	-	N1R/p28 family
**170**	611	I	fpv162/pepv172/fep165/cnpv223/tkpv015	603	89/88/86/32/30	-	Ankyrin repeat family
**171**	249	I	pepv173/fep166/fpv163/cnpv159/tkpv050	256	94/90/76/33/33	-	N1R/p28 family
**172**	741	I	pepv174/fpv162/cnpv223/fep167/tkpv120	741	95/33/35/32/30	-	Ankyrin repeat family
**173**	379	I	pepv175/fep168/fpv164/cnpv235	386	90/90/67/31	-	Hypothetical protein
**174**	300	I	cnpv236/Elephantulus edwardii	324	83/72	F4L	Ribonucleotide reductase small subunit
**175**<br>**176**<br>**177**	134<br>93<br>71	F<br>F<br>F	cnpv237	441	-	C9L	Ankyrin repeat family
**178**	225	I	fpv165/pepv177/fep169/tkpv121/cnpv238	225	98/96/95/91/91	A2L	Late transcription factor VLTF-3
**179**	72	I	pepv178/fpv166/cnpv239/fep170???	72	100/96/73	A2.5L	Virus redox protein
**180**	660	I	pepv179/fep171/fpv167/cnpv240/tkpv122	657	99/99/98/84/82	A3L	Virion core protein P4b
**181**	265	I	pepv180/fep172/fpv168/cnpv241/tkpv123	255	83/80/60/48/43	A4L	Immunodominant virion protein
**182**	169	I	pepv181/fep173/fpv169/cnpv242/tkpv124	169	100/99/98/75/77	A5R	RNA pol subunit RP019
**183**	374	I	pepv182/fep174/fpv170/cnpv243/tkpv125	374	98/98/95/68/63	A6L	Hypothetical protein
**184**	709	I	pepv183/fep175/fpv171/cnpv244/tkpv126	709	99/99/97/88/85	A7L	Early transcription factor large subunit VETF-L
**185**	301	I	pepv184/fep176/fpv172/cnpv245/tkpv127	301	99/99/99/79/75	A8R	Intermediate transcription factor VITF-3
**186**	76	I	fpv173/fep177/pepv185/cnpv246	76	88/87/87/57	A9L	Hypothetical protein
**187**	891	I	fep178/pepv186/fpv174/cnpv247/tkpv128	891	98/98/96/71/67	A10L	Virion core protein P4a
**188**	272	I	pepv187/fep179/fpv175/tkpv129/cnpv248	272	99/99/97/63/66	A11R	Hypothetical protein
**189**	175	I	pepv188/fep180 /fpv176/cnpv249/tkpv130	175	99/98/91/61/58	A12L	Virion core protein
**190**	70	I	pepv189/fpv177	70	97/85	-	Hypothetical protein
**191**	71	I	pepv190/fep182/fpv178/cnpv251	71	97/96/87/56	A13L	Virion membrane protein
**192**	91	I	pepv191/fep183/fpv179/cnpv252	91	100/99/96/61	A14L	Virion envelope protein
**193**	53	I	pepv192/fpv179.1/fep184	53	100/98	a14.5	Virion envelope protein
**194**	97	I	fep185/fpv180/pepv193/cnpv254	97	98/95/95/60	A15L	Hypothetical protein
**195**	368	I	fep186/pepv194/fpv181/cnpv255/tkpv131	369	94/94/92/77/75	A16L	Putative myristoylated membrane protein
**196**	198	I	fep187/pepv195/fpv182/cnpv256/tkpv132	198	99/99/98/79/64	A17L	Phosphorylated virion membrane protein
**197**	462	I	pepv196/fep188/fpv183/cnpv257/tkpv133	462	99/98/97/84/76	A18R	DNA helicase
**198**	88	I	pepv197/fep189/fpv184/cnpv258/tkpv134	88	100/98/95/68/57	A19L	Zinc finger like protein
**199**	113	I	fpv186/fep190/pepv198/cnpv260/tkpv135	113	97/95/94/85/80	A21L	Hypothetical protein
**200**	432	I	pepv199/fep191/fpv185/cnpv259/tkpv136	432	99/99/99/98/56	A20R	Processivity factor
**201**	161	I	pepv200/fep192/fpv187/cnpv261/tkpv137	161	99/97/91/72/72	A22R	Holliday junction resolvase
**202**	383	I	pepv201/fep193/fpv188/cnpv262/tkpv138	383	98/98/96/73/72	A23R	Intermediate transcription factor VITF-3
**203**	1157	I	fep194/pepv202/fpv189/cnpv263/tkpv139	1157	99/99/98/91/91	A24R	RNA pol subunit RPO132
**204**	612	I	fep195/pepv203/fpv190/cnpv264/tkpv140	608	97/97/89/68/62	A25L	A-type inclusion protein
**205**	474	I	fep196/pepv204/fpv191/cnpv265/tkpv141	472	97/97/92/78/48	A26L	A-type inclusion protein
**206**	140	I	pepv205/fpv192/cnpv266/hgpv192/tkpv142	140	99/96/84/84/76	A28L	Hypothetical protein
**207**	302	I	pepv206/fep198/fpv193/hgpv193/cnpv267/tkpv143	302	97/95/91/65/64/61	A29L	RNA pol subunit RPO35
**208**	74	I	fep199/pepv207/fpv194/tkpv143/cnpv268	74	100/100/98/71/83	A30L	Hypothetical protein
**209**	38	I	pepv208/fep200/fpv194.1	38	97/95/84	A30.5L	A30.5L orthologue
**210**	113	I	pepv209/fep201/fpv195/cnpv269	113	100/97/90/64	A31R	Hypothetical protein
**211**	120	I	fep202/pepv210/fpv196/cnpv270	120	94/94/83/42		Hypothetical protein

**212**	304	I	pepv211/fep203/fpv197/cp197L (vultur gryphus poxvirus)/cnpv271/tkpv145	304	98/97/92/78/75/67	A32L	Virion assembly protein
**213**	173	I	pepv212/fep204/fpv198/cnpv272/tkpv146	173	97/97/93/66/57	A34R	C-type lectin family
**214**	220	I	pepv213/fpv199/tkpv147	220	93/81/41	-	V-type Ig Domain
**215**	263	I	pepv214/fpv200/tkpv148	263	95/85	-	V-type Ig domain
**216**	277	I	fep207/pepv215/fpv201/cnpv273/tkpv149	277	96/95/91/45	-	Hypothetical protein
**217**	285	I	fep208/pepv216/fpv203/cnpv274/tkpv150	285	98/97/84/51	-	Tyrosine protein kinase
**218**	342	I	fep209/pepv217/fpv204/cnpv275	342	97/96/92/54	C12L	Serpin family
**219**	220	I	pepv218/fep210/fpv205/cnpv276/tkpv151	220	95/93/85/38	-	Hypothetical protein
**220**	308	I	pepv219/fep211/fpv206/cnpv277	308	99/98/89/67	-	G-protein-coupled receptor family
**221**	92	I	pepv220/fep212/fpv207/cnpv278	92	96/95/90/66	-	Hypothetical protein
**222**	179	I	cnpv279/fpv072	169	52/34	-	β-NGF-like family
**223**	137	I	cnvp280/pepv162/fpv152	130	41/28/28	-	HT motif family
**224**	212	I	pepv222/fep214/cnpv281/fpv208/tkpv152	193	98/96/47/67	-	Hypothetical protein
**225**	145	I	pepv223/fep215/fpv209/cnpv282	137	99/91/83/45	-	HT motif family
**226**	108	I	cnpv283	111	50	-	CC chemokine family
**227**	157	E 5'	pepv225/fep216/fpv211/cnpv285/tkpv153	125	96/93/81/43	C11R	Epidermal Growth Factor-like protein
**228**	303	I	fep217/fpv212/pepv226/cnpv286/tkpv154	303	95/92/86/55	B1R	Serine/threonine protein kinase
**229**	162	I	fep218/pepv227/fpv213/cnpv287/tkpv155	162	97/96/91/54	-	Hypothetical protein
**230**	124	I	fep219/pepv228/fpv214/cnpv289/tkpv156	125	91/93/85/47	-	Putative 13.7 kDa protein
**231**	74	I	pepv229/fep220???/fpv215/cnpv290	74	100/95/81	-	Hypothetical protein
**232**	174	F	brevican core protein Xenopus Silurana tropicalis (fep221 fragment)	1146	38/23	-	Hypothetical brevican core protein
**233**	294	I	pepv230/fep222/fpv216/cnpv293/tkpv157	294	96/95/88/37	C18L	Ankyrin repeat family
**234**	143	I	fep223/pepv231/Tanapox 67R (178aa)	143	98/96/36	C7L	67R Tanapox host range protein
**235**<br>**236**	237<br>59	T<br>F	fpv217	328	-	-	Hypothetical protein
**237**	474	I	pepv233/fpv218/cnpv295	474	94/84/37	B4R	Ankyrin repeat family
**238**	442	I	fep226/fpv219/pepv234/cnpv296/tkpv161	440	95/95/87/46	B4R	Ankyrin repeat family
**239**	117	I	fpv220	117	100	-	Hypothetical protein
**240**	183	I	pepv235/fep227/fpv221	183	98/96/90	A47L	Hypothetical protein
**241**	745	I	fep228/pepv236/fpv222/cnpv297	747	84/83/79/36	B4R	Ankyrin repeat family
**242**	292	I	pepv240/fpv226/fep231/cnpv299/tkpv154	293	91/89/91/57/43	B1R	Serine/threonine protein kinase
**243**	361	I	pepv241/fep232/fpv227/cnpv300/tkpv162	361	96/96/88/46	-	Ankyrin repeat family
**244**<br>**245**<br>**246**	204<br>239<br>297	T<br>F<br>F	fpv228/trichomonas vaginalis DUF3447/pepv275/cnpv044	525	-	-	Ankyrin repeat family
**247**	502	I	fep248/pepv253/cnpv301/fpv233	503	97/95/39/30	-	Ankyrin repeat family
**248**	348	I	cnpv022/fpv010/fep011/pepv011	358	37/36/36/35	C12L	Serpin family
**249**	187	I	fep236/pepv246/fpv229/cnpv302	185	97/93/82/33	-	Hypothetical A47L-like protein
**250**	502	I	pepv247/fpv231/cnpv303	514	96/87/50	B4R	Ankyrin repeat family
**251**	489	I	pepv248/fep238/fpv232/cnpv304	481	96/96/86/46	-	Ankyrin repeat family
**252**	503	I	fep239/pepv249/fpv233	502	96/90/96	-	Ankyrin repeat family
**253**	430	I	pepv250/fpv234	422	95/86	-	Ankyrin repeat family

**254**	148	I	pepv251/fpv235/cnpv029	148	91/71/32	A40R	C-type lectin family
**255**	275	I	pepv252/fep241/fpv236/cnpv305	274	94/93/80/36	-	N1R/p28 family
**256**	72	-	No significant homology	-	-	-	Hypothetical P-type ATPase
**257**	163	I	pepv254/fpv239/cnpv307/tkpv165	163	94/83/46	A40R	C-type lectin family
**258**	88	E 5’	fpv238	61	57	-	Hypothetical protein
**259**	411	I	fep244/pepv255/fpv240/cnpv308	411	90/86/83/35	-	Ankyrin repeat family
**260**	185	I	pepv257/fpv241/cnpv309	185	96/90/46	-	Ankyrin repeat family
**261**<br>**262**	159<br>317	T<br>F	cnpv310/fpv246/pepv266/fep252	592	-	-	Ankyrin repeat family
**263**	88	F	cnpv312	166			Hypothetical protein
**264**	210	E 5’	cnpv312	166	36	-	Hypothetical protein
**265**	643	I	pepv259/fep246/cnpv314/fpv242	643	97/97/56/90	-	Ankyrin repeat family
**266**	192	I	fep258/pepv264/cnpv309/fpv241	192	95/95/42/33	-	Ankyrin repeat family
**267**	463	I	fep006/pepv273/cnpv320/fpv017	467	33/32/32/30	-	V-type Ig domain
**268**	667	I	fep260/fpv244/cnpv009/pepv010	667	91/81/79/31	-	Ankyrin repeat family
**269**	444	I	pepv264/fpv245/cnpv020	440	92/81/45	-	Ankyrin repeat family
**270**<br>**271**	254<br>235	T<br>F	cnpv014/pepv262/fep256/fpv017	490	-	-	V-type Ig domain
**272**	584	I	fep264/pepv272/fpv246/cnpv011	585	93/92/89/46	-	Ankyrin repeat family
**273**	124	I	fep253/pepv267/fpv247/cnpv321	122	96/95/85/60	-	EFc family
**274**	149	I	fep254/pepv268/fpv248/cnpv218	149	87/88/86/43	-	N1R/p28 family
**275**	98	F	trichomonas vaginalis G3 ankyrin repeat protein	732	-	-	Ankyrin repeat family
**276**<br>**277**	288<br>544	T<br>F	fpv162/fep167/pepv175/cnpv150	603	40/38/37/32	-	Ankyrin repeat family
**278**	108	F	pepv269	628	88	B20R	Ankyrin repeat family
**279**	110	I	fep267/fpv255	107	89/71	-	Hypothetical protein
**280**	124	I	pepv271/fep256/fpv258/cnpv035	124	86/86/71/29	A40R	C-type lectin family
**281**	169	F	pepv275/fpv162/cnpv021/fep167	628	-	-	Ankyrin repeat family
**282**	508	I	pepv009/fep008/cnpv015	508	93/93/45	-	Ankyrin repeat family
**283**	201	I	fep126 /cnpv012	203	47/37	A47L	Hypothetical protein
**284**	419	I	fep007/pepv008/fpv006/cnpv214	410	95/95/85/43	C10L	C4L/C10L family
**285**	140	I	pepv007/fpv250	139	93/80	A40R	US ORF2

* I=intact, F=fragmented, T=truncated and E=extended relative to the closest orthólouge
